# NEK2 is essential for spindle assembly checkpoint regulation and cytoskeleton organization in porcine oocyte meiotic maturation

**DOI:** 10.1007/s11010-026-05674-z

**Published:** 2026-08-04

**Authors:** Se-Been Jeon, Pil-Soo Jeong, Hyo-Gu Kang, Ji Hyeon Yun, Se-Yeon Eom, Na-Gyeom Oh, Yeounsun Oh, Seung Hwan Lee, Sun-Uk Kim, Seong-Keun Cho, Bo-Woong Sim

**Affiliations:** 1 Futuristic Animal Resource & Research Center (FARRC), Korea Research Institute of Bioscience and Biotechnology (KRIBB), Cheongju, 28116 Republic of Korea; 2 Department of Animal Science, College of Natural Resources & Life Science, Pusan National University, Miryang, 50463 Republic of Korea; 3 Department of Animal Science and Biotechnology, College of Agriculture and Life Science, Chungnam National University, Daejeon, 34134 Republic of Korea; 4 Department of Life Science, Chung-Ang University, Seoul, 6974 Republic of Korea; 5 Department of Functional Genomics, University of Science and Technology, Daejeon, 34113 Republic of Korea; 6 Department of Animal Science, Life and Industry Convergence Research Institute (RICRI), College of Natural Resources & Life Science, Pusan National University, Miryang, 50463 Republic of Korea

**Keywords:** NEK2, Oocyte meiosis maturation, Cell cycle, Kinetochore-microtubule attachment, Spindle assembly checkpoint, Cytoskeleton

## Abstract

NIMA-related kinase 2 (NEK2) is a serine/threonine kinase that plays crucial roles in cellular events such as centrosome separation, cell cycle progression, spindle assembly checkpoint (SAC) regulation, and microtubule stabilization. However, its regulatory function during oocyte meiotic maturation remains unclear. In this study, NEK2 activity was inhibited using the specific inhibitor MBM-55 to evaluate its role in oocyte meiotic maturation and early embryonic development. NEK2 depletion impaired multiple indicators associated with oocyte maturation and decreased the maturation rate. Mechanistic analysis suggested that NEK2 inhibition altered spindle organization and chromosome alignment, which may be associated with impaired kinetochore–microtubule (K–MT) interactions and altered SAC-associated signaling, collectively contributing to MI-stage arrest and increased aneuploidy. In addition, NEK2 deficiency impaired the actin network, reducing spindle migration, preventing the efficient repair of accumulated DNA damage and ultimately leading to apoptosis. NEK2 inhibition in oocytes reduced subsequent embryonic developmental competence, leading to a lower blastocyst formation rate. These findings demonstrate that NEK2 is a critical regulator of porcine oocyte meiotic maturation through its roles in SAC regulation, K–MT attachment, cytoskeletal dynamics, and the DNA damage response.

**Supplementary Information:**

The online version contains supplementary material available at https://doi.org/10.1007/s11010-026-05674-z.

## Introduction

Mammalian oocyte maturation is a complex process regulated by multiple factors that collectively determine oocyte quality. Oocytes generate haploid gametes that represent their fully mature state, and the successful completion of meiosis is essential for early embryonic development [1]. Fully grown oocytes within the ovary are arrested at the germinal vesicle (GV) stage until meiosis resumes upon hormonal stimulation, leading to germinal vesicle breakdown (GVBD) and chromatin condensation. During metaphase I (MI), chromosomes align along the equatorial plate, and asymmetric cytokinesis occurs with the support of a microtubule-based spindle and actin network. The oocyte is then arrested at metaphase II (MII) until fertilization occurs [2].

Unlike in somatic cells, chromosome movement and spindle assembly in oocytes are regulated by acentriolar microtubule-organizing centers (aMTOCs), which are enriched with centrosomal proteins [3]. Thus, aMTOCs play a key role in microtubule nucleation and spindle pole organization during oocyte maturation and early embryogenesis [4]. Proper centrosome function ensures the stability of kinetochore–microtubule (K–MT) attachments, whereas centrosomal defects disrupt spindle microtubules or microtubule motor function, thereby activating the spindle assembly checkpoint (SAC) [5, 6]. The SAC, which includes Bub and Mad family proteins, safeguards accurate meiotic progression by preventing abnormal chromosome segregation [7]. Errors in this process can lead to improper chromosome segregation, DNA damage, aneuploidy, and disruption of normal cell cycle progression [8]. The centrosome is a critical component of the cell cycle regulatory machinery, and its associated kinases are essential for the execution and control of cell cycle progression [9].

The failure of checkpoint mechanisms can lead to excessive accumulation of DNA damage. Ataxia telangiectasia mutated (ATM) recognizes DNA lesions via the MRE11–RAD50–NBS1 complex and subsequently induces H2AX phosphorylation [10]. Phosphorylated H2AX at Ser139 (γ-H2AX) facilitates homologous recombination (HR) and non-homologous end joining (NHEJ) repair pathways by stabilizing or recruiting repair effector proteins [11]. However, when DNA repair mechanisms fail, excessive DNA damage triggers cellular responses such as cell cycle arrest or apoptosis [12].

Never in mitosis A (NIMA) is a serine/threonine kinase first identified in *Aspergillus*. In mammals, 11 members of the NIMA-related kinase family have been characterized, among which NIMA-related kinase 2 (NEK2) shares the highest sequence similarity with NIMA in the human genome [13]. As the most extensively studied member of this family, NEK2 plays pivotal roles in cell differentiation and homeostasis, including centrosome separation, microtubule stabilization, cell cycle regulation, and the DNA damage response [14, 15]. Aberrant expression of NEK2 has been shown to induce centrosome fragmentation, abnormal spindle formation, and chromosome segregation errors, ultimately leading to aneuploidy [16]. In mouse oocytes, NEK2 inhibition has been reported to cause spindle abnormalities and impaired meiotic maturation [4]. NEK2 is a multifunctional regulator of the DNA damage response and cell cycle progression, and it participates in multiple signaling pathways [17]. In porcine embryos, NEK2 inhibition has been associated with elevated DNA damage and induced apoptosis caused by defects in the DNA repair machinery [18]. Although NEK2 has been implicated in essential cellular processes across various species and cell types, its specific roles in porcine oocyte maturation remain poorly understood.

Therefore, we investigated the role of NEK2 in porcine oocyte maturation by evaluating maturation-related markers, checkpoint activity, cytoskeletal organization, DNA damage, and subsequent embryonic development. Our findings provide new insights into the regulatory mechanisms of NEK2 and its potential as a candidate gene involved in porcine oocyte maturation and developmental competence.

## Materials and methods

### Chemicals

All regents and chemicals were purchased from Sigma Aldrich (St. Louis, MO, USA), unless otherwise stated.

### In vitro maturation (IVM)

Ovaries were obtained from a slaughterhouse and placed in 0.9% physiological saline supplemented 50 µg/mL streptomycin sulfate and 75 µg/mL benzylpenicillin potassium G at 38.5 °C. Cumulus oocyte complexes (COCs) were aspirated from 3–6-mm follicles using an 18-gauge needle. Three or more layers of compact cumulus cells were selected and washed with 0.9% saline supplemented with 0.1% bovine serum albumin (BSA). COCs were incubated for 22 h at 38.5 °C in 5% CO_2_ in IVM medium composed of tissue culture medium 199 supplemented with 10% porcine follicular fluid, 25 µM B-mercaptoethanol, 10 ng/mL epidermal growth factor, 0.57 mM cysteine, 10 IU/mL pregnant mare serum gonadotropin (HOR-272; Prospec, Rehovot, Israel), and 10 IU/mL human chorionic gonadotropin (HOR-250; Prospec), and then incubated for a further 22 h at 38.5 °C in 5% CO_2_ in hormone-free medium.

### Chemical treatment

We used the NEK2-specific inhibitor MBM-55 (MBM; HY-101029; MedChemExpress, Monmouth Junction, NJ, USA), at final concentrations of 0, 6, 8, and 10 µM in IVM medium. MBM was dissolved in dimethyl sulfoxide, with the final concentration of the latter maintained below 0.1%.

### Analysis of cumulus cell expansion and oocyte nuclear maturation

After 44 h of IVM, cumulus cell expansion was evaluated based on the morphological characteristics of COCs and classified as grade 1 (compact with minimal observable expansion), grade 2 (partial expansion limited to outer layers), grade 3 (expansion of most cumulus layers except the corona radiata), or grade 4 (complete expansion of all layers). Then, cumulus cells were removed by gentle pipetting in 0.1% hyaluronidase, and the denuded oocytes were examined under a stereomicroscope (Nikon, Tokyo, Japan) to assess nuclear maturation status. Oocytes were categorized as MII (presence of first polar body), immature (absence of first polar body), or degenerate.

### Parthenogenetic activation and in vitro culture (IVC)

After removing cumulus cells from oocytes using 0.1% hyaluronidase, MII oocytes were selected for parthenogenetic activation. The MII oocytes were cultured in Dulbecco’s phosphate-buffered saline (DPBS; Thermo Fisher Scientific, Waltham, MA, USA) supplemented with 60 µg/mL gentamicin sulfate, 75 µg/mL streptomycin sulfate, and 4 mg/mL BSA, and then exposed to 15 µM ionomycin for 5 min in the dark. The activated oocytes were then transferred to porcine zygote medium-3 (IVC medium) containing 4 mg/mL BSA, supplemented with 5 µg/mL cytochalasin B and 2 mM 6-dimethylaminopurine, and incubated at 38.5 °C under a 5% CO_2_ atmosphere for 4 h. Then, the oocytes were washed and transferred to fresh IVC medium and further cultured at 38.5 °C under 5% CO_2_ for 6 days.

### Immunocytochemistry

Fixed samples were washed with DPBS containing 0.1% polyvinyl alcohol (PVA-PBS), and then permeabilized with DPBS containing 1% (v/v) Triton X-100 for 1 h at room temperature (RT). Following permeabilization, the samples were washed with PVA-PBS and transferred to a blocking solution consisting of DPBS supplemented with 0.05% (v/v) Tween-20 and 2 mg/mL BSA, and incubated at RT for 1 h. For p-ATM and CDX2 staining, blocking was performed using PVA-PBS supplemented with 1 mg/mL BSA; subsequent washes were performed with PVA-PBS. After blocking, the samples were incubated overnight at 4 °C with primary antibodies targeting NEK2 (1:500; ab227958; Abcam, Cambridge, UK), BMP15 (1:200; PA5-34401; Invitrogen, Carlsbad, CA, USA), p-ATM (1:100; 5883 S; Cell Signaling Technology, Danvers, MA, USA), γ-H2AX (1:200; 2577 S; Cell Signaling Technology), BUBR1 (1:100; ab254326; Abcam), α-tubulin (1:500; ab64503; Abcam), γ-tubulin (1:200; ab179503; Abcam), Ac-tubulin (1:200), and CDX2 (1:200; D11D10; Cell Signaling Technology). The next day, the samples were washed and incubated in the same blocking solution used previously at RT for 1 h, followed by incubation with appropriately diluted secondary antibodies (1:200; A-11008, A-11012, A-11029, and A-11032; Thermo Fisher Scientific) at RT for 1 h. Finally, the samples were washed and mounted on glass slides using VectaShield mounting medium containing 4′,6-diamidino-2-phenylindole (DAPI; Vector Laboratories, Newark, CA, USA). Fluorescence signal intensity was observed using a fluorescence microscope (DMi8; Leica Microsystems, Wetzlar, Germany) and analyzed with ImageJ software v1.47 (National Institutes of Health, Bethesda, MD, USA).

### Western blotting

Western blotting was performed as previously described [19]. Briefly, samples were lysed using an extraction buffer containing a protease inhibitor cocktail and heated at 100 °C for 10 min. Protein extracts were separated by 10% sodium dodecyl sulfate–polyacrylamide gel electrophoresis and transferred onto nitrocellulose membranes. After blocking in Tris-buffered saline with 0.1% Tween-20 (TBST) containing 5 mg/mL BSA, the membranes were incubated overnight at 4 °C with primary antibodies against NEK2 (1:200; ab227958; Abcam), p-ERK (1:200; 9101; Cell Signaling Technology), T-ERK (1:200; 9102; Cell Signaling Technology), and GAPDH (1:1000; LF-PA0212; AbFrontier, Seoul, Korea). Following washes with TBST, membranes were re-blocked in 5% skim milk and incubated overnight at 4 °C with horseradish protein-conjugated secondary antibodies (goat anti-rabbit or goat anti-mouse IgG, 1:5000 dilution). Signal detection was performed using the SuperSignal West Pico Plus Chemiluminescent Substrate (34580; Thermo Fisher Scientific). Band intensities were quantified using ImageJ software.

### Quantitative reverse-transcription polymerase chain reaction (qRT-PCR)

Total RNA was extracted from samples using a Dynabeads mRNA Direct Kit (Invitrogen), and cDNA was synthesized with a Prime Script RT Reagent Kit with gDNA Eraser (Takara Bio, Shiga, Japan). The qRT-PCR conditions were as follows: 95 °C for 5 min, followed by 40 cycles of 95 °C for 20 s, and 60 °C for 20 s. Experiments were performed using the Mx3000P qPCR system (Agilent Technologies, Santa Clara, CA, USA) and SYBR Premix Ex Taq (Takara Bio). GAPDH was used as the internal control, and gene expression was quantified in terms of fold changes using the Eq. 2^–(SΔCT–CΔCT)^. The primer sequences are listed in Supplementary Table 1.

### Flow cytometry

Cumulus cells were collected and centrifuged at 2,000 rpm for 3 min, then fixed in 70% ethanol. After washing with DPBS, the cells were resuspended in 500 µL of 50 µg/mL propidium iodide solution and incubated for 30 min in the dark. The cells were subsequently washed with DPBS and analyzed using a flow cytometer (BD Biosciences, Franklin Lakes, NJ, USA). Data analysis was performed using FlowJo software (FlowJo, Ashland, OR, USA). During flow cytometry analysis, cell debris was excluded and single-cell populations were gated prior to analysis. Cell-cycle phase distributions (G0/G1, S, and G2/M) were determined by manually defining gates according to DNA content histograms generated from propidium iodide fluorescence intensity.

### Annexin-V

Early apoptosis was assessed using the Annexin V–FITC Apoptosis Detection Kit (BD Biosciences). Samples with the zona pellucida removed were fixed, washed with PVA-PBS, and stained in 45 µL of binding buffer containing 5 µL of annexin V–fluorescein isothiocyanate for 30 min at 38.5 °C. After staining, the samples were again washed with PVA-PBS and immediately examined using a laser-scanning confocal fluorescence microscope (LSM700; Zeiss, Oberkochen, Germany).

### Chromosome spreading

Samples with the zona pellucida removed were fixed on glass slides in 1% paraformaldehyde in distilled water (pH 9.2) containing 0.15% Triton X-100 and 3 mM dithiothreitol for 2 h at RT. After fixation and air-drying, the samples were incubated with anti-BUBR1 primary antibody (1:100; ab254326; Abcam) overnight at 4 °C, followed by incubation with Alexa Fluor 488-conjugated goat anti-rabbit secondary antibody for 1 h at RT. Chromosomes were counterstained with DAPI (Vector Laboratories), and samples were examined using a laser-scanning confocal fluorescence microscope (LSM700; Carl Zeiss, Oberkochen, Germany).

### Assessment of actin filaments and transzonal projections (TZPs)

For the analysis of actin filaments and TZPs, permeabilized samples were washed with PVA-PBS and then blocked with a solution consisting of DPBS supplemented with 0.05% (v/v) Tween-20 and 2 mg/mL BSA at RT for 1 h. After blocking, the samples were stored in the blocking solution and subsequently stained with 10 µg/mL phalloidin-tetramethylrhodamine B isothiocyanate at RT for 2 h. Following staining, the samples were washed, mounted with DAPI (Vector Laboratories) on slide glasses, and analyzed using ImageJ software to evaluate actin filament and TZPs signal intensities.

### TdT-mediated dUTP nick-end labeling (TUNEL) assays

Apoptosis was detected using the In Situ Cell Death Detection Kit (Roche, Basel, Switzerland). Fixed samples were washed with PVA-PBS and permeabilized with 1% (v/v) Triton X-100 for 1 h at RT. The samples were again washed with PVA-PBS and incubated at 38.5 °C in TUNEL assay solution for 1 h. After staining, the samples were washed with PVA-PBS and mounted on glass slides with DAPI (Vector Laboratories). Apoptotic cells were counted using a fluorescence microscope (DMi8; Leica Microsystems).

### Statistical analyses

Unless otherwise specified, all experiments were performed with three biological replicates, yielding consistent results. Data are presented as mean ± standard error of the mean. Comparisons between two groups were conducted using Student’s t-test, and comparisons among three or more groups were conducted using one-way analysis of variance followed by Tukey–Kramer post hoc tests. Significant differences were evaluated at a threshold of *P* < 0.05. All analyses were performed using SigmaStat software (Systat Software, San Jose, CA, USA).

## Results

### NEK2 is essential for porcine oocyte meiotic maturation

**Fig. 1:**
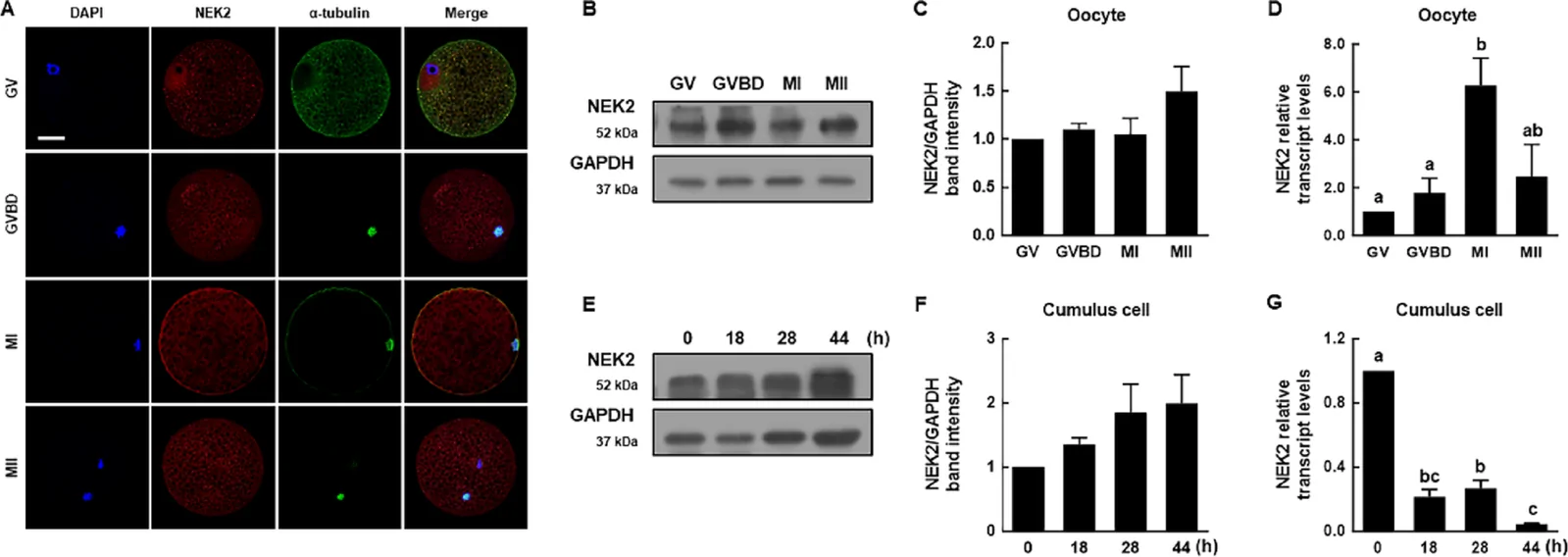
Expression and subcellular localization of NIMA-related kinase 2 (NEK2) during meiotic progression in porcine oocytes. **A** Representative images of different meiotic stages: germinal vesicle, germinal vesicle breakdown, metaphase I (MI), and metaphase II (MII). Scale bar = 50 μm. **B** Western blot analysis and **C** quantification of NEK2 protein levels in oocytes in the indicated groups (*n* = 3 per group). **D** Quantitative reverse-transcription polymerase chain reaction (qRT-PCR) analysis of NEK2 transcript levels in oocytes from the indicated groups (*n* = 3 per group). **E** Western blot analysis and **F** quantification of NEK2 protein levels in cumulus cells in the indicated groups (*n* = 3 per group). **G** qRT-PCR analysis of NEK2 transcript levels in cumulus cells in the indicated groups (*n* = 3 per group). Data are means ± standard error of the mean (SEM) for three independent experiments. Different letters indicate significant differences (*P* < 0.05)

To investigate the effects of NEK2 on porcine oocyte maturation, we performed immunostaining, Western blotting, and qRT-PCR at various meiotic stages. Oocytes were collected at 0, 18, 28, and 44 h of culture, which corresponded to the GV, GVBD, MI, and MII stages, respectively. NEK2 was broadly localized throughout the cytoplasm, including the nuclear region, during the GV to MII stages of oocyte maturation (Fig. 1A). In oocytes, NEK2 protein levels remained relatively constant from the GV to MII stages (Fig. 1B, C), whereas mRNA levels peaked significantly at the MI stage (Fig. 1D). Cumulus cells were collected at 0, 18, 28, and 44 h after culture, depending on the oocyte maturation stage. In cumulus cells, NEK2 protein expression remained relatively constant during meiosis (Fig. 1E, F), whereas mRNA levels gradually decreased as meiosis progressed (Fig. 1G). These patterns of NEK2 expression and localization suggest a critical role for NEK2 in the regulation of oocyte meiotic maturation.

### NEK2 is indispensable for porcine oocyte meiotic progression

**Fig. 2:**
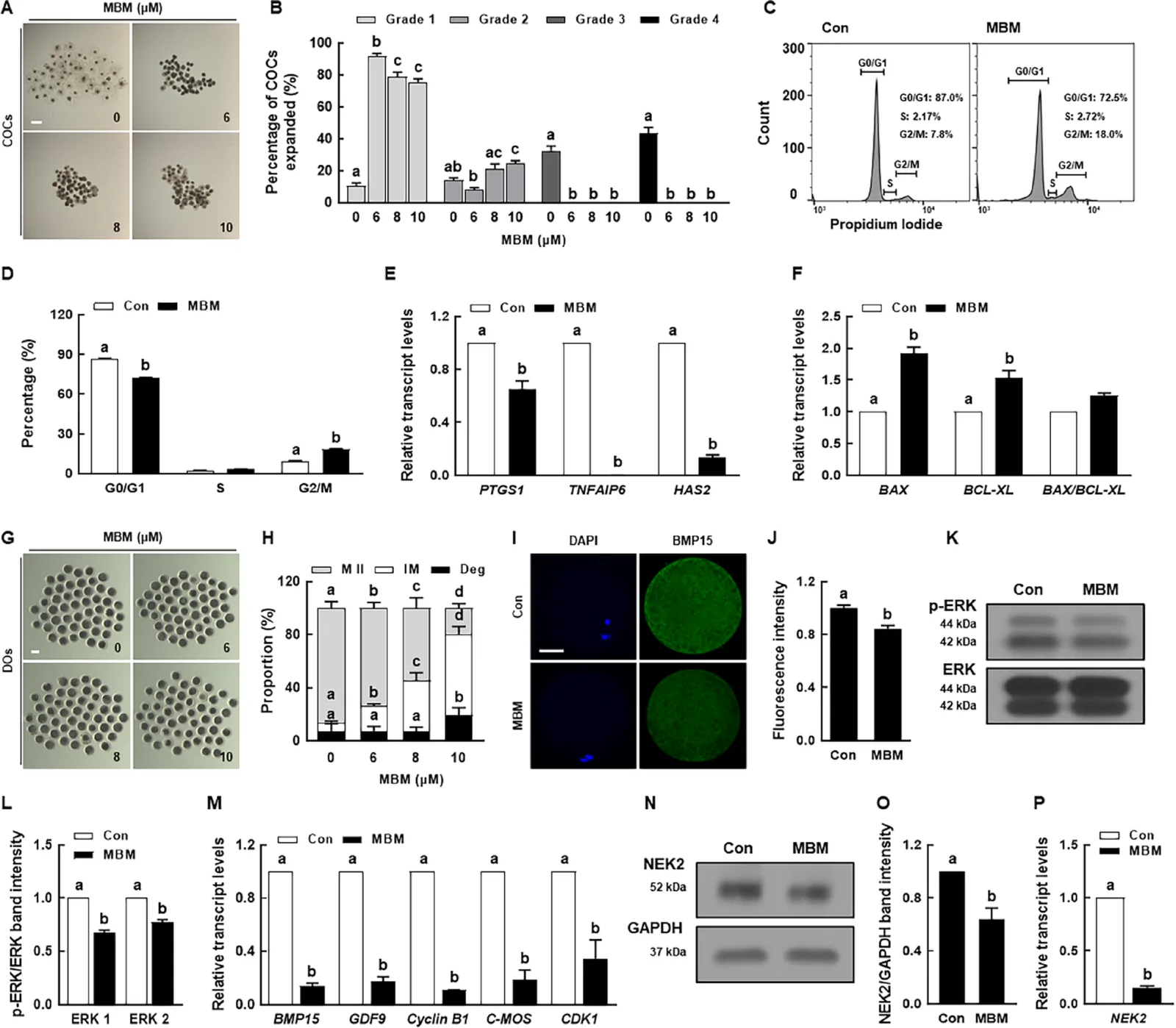
Effects of NEK2 inhibition on porcine oocyte meiotic progression. (A) Representative images of cumulus cell complexes and (B) proportions of cumulus cell expansion in the indicated groups (*n* = 250 per group) Scale bar = 200 μm. (C) Representative flow cytometry plots and (D) proportions of cell cycle phases in cumulus cells in the indicated groups (*n* = 3 per group). (E) qRT-PCR analysis of transcript levels of cumulus expansion-related genes (*PTGS1*, *TNFAIP6*, and *HAS2*) in cumulus cells in the indicated groups (*n* = 3 per group). (F) qRT-PCR analysis of transcript levels of apoptosis-related genes (*BAX* and *BCL-XL*) in cumulus cells in the indicated groups (*n* = 3 per group). (G) Representative images of denuded oocytes and (H) proportions of different nuclear maturation stages in the indicated groups (*n* = 200 per group) Scale bar = 100 μm. (I) Representative images and (J) fluorescence intensity of BMP15 in oocytes (*n* = 61 per group) Scale bar = 50 μm. (K) Western blot analysis and (L) quantification of p-ERK and ERK protein levels in oocytes in the indicated groups (*n* = 3 per group). (M) qRT-PCR analysis of transcript levels of oocyte competence-related genes (*BMP15*, *GDF9*, *Cyclin B1*, *C-MOS*, and *CDK1*) in oocytes in the indicated groups (*n* = 3 per group). (N) Western blot analysis and (O) quantification of NEK2 protein levels in oocytes in the indicated groups (*n* = 3 per group). (P) qRT-PCR analysis of transcript levels of NEK2 in oocytes in the indicated groups (*n* = 3 per group). Data are mean ± SEM for three independent experiments. Different letters indicate significant differences (*P* < 0.05)

To investigate the effects of NEK2 on porcine oocyte meiotic progression, COCs were cultured in IVM medium supplemented with various concentrations (0, 6, 8, and 10 µM) of MBM, an NEK2-specific inhibitor. NEK2 inhibition significantly impaired cumulus expansion, particularly at grades 1 and 4 (Fig. 2A, B). Flow cytometry was performed to determine whether reduced cumulus expansion following NEK2 inhibition affected the cell cycle of cumulus cells. Treatment with 8 µM MBM induced G2/M cell cycle arrest, as indicated by a decrease in the G0/G1 phase population and an increase in the G2/M phase population compared with controls (Fig. 2C, D). Furthermore, 8 µM MBM treatment suppressed the expression of cumulus expansion-related genes (*PTGS1*, *TNFAIP6*, and *HAS2*) and upregulated that of apoptosis-related genes (*BAX* and *BCL-XL*), as reflected by an increased *BAX*/*BCL-XL* ratio (Fig. 2E, F). NEK2 inhibition also decreased the MII rate compared with controls and increased the proportion of immature oocytes in a dose-dependent manner (Fig. 2G, H and Supplementary Table 2). Treatment with 8 µM MBM significantly reduced the protein levels of BMP15 and ERK, as well as the expression of oocyte maturation-related genes (*BMP15*, *GDF9*, *Cyclin B1*, *C-MOS*, and *CDK1*) compared with controls (Fig. 2I–M). Consistently, MBM treatment also reduced NEK2 protein and mRNA levels relative to controls (Fig. 2N–P). These findings indicate that NEK2 plays an essential role in regulating meiotic progression and maturation in porcine oocytes.

### NEK2 regulates meiotic progression and SCA integrity during porcine oocyte maturation

**Fig. 3:**
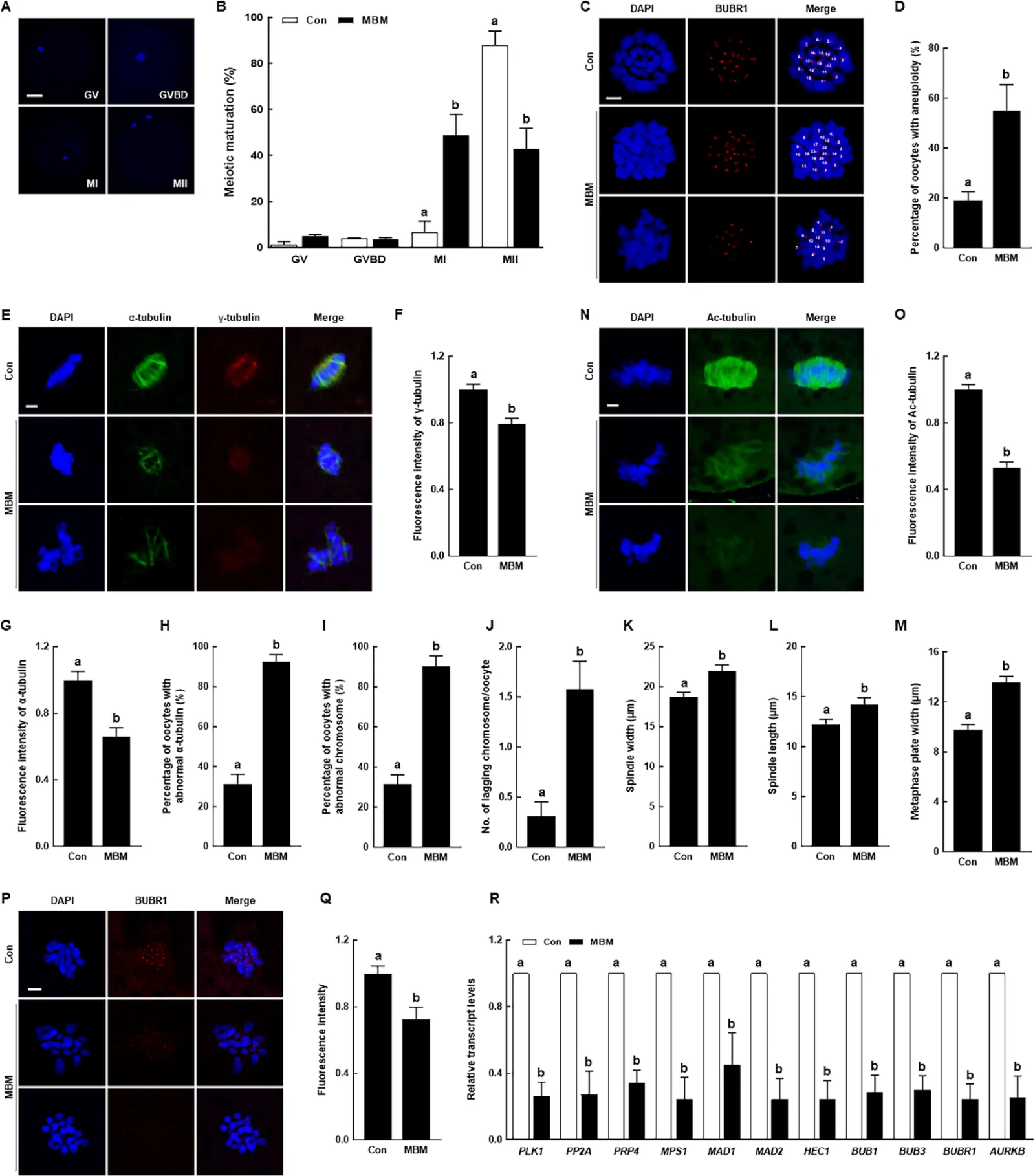
Effects of NEK2 inhibition on cell cycle and spindle assembly checkpoint during oocyte meiotic progression. **A** Representative images of different nuclear maturation stages and **B** proportions of meiotic maturation in the indicated groups (control [Con]: *n* = 148, MBM-55 treatment [MBM]: *n* = 142). Scale bar = 50 μm. **C** Representative images and **D** percentage of aneuploidy in MII oocytes in the indicated groups (Con: *n* = 39, MBM: *n* = 44). Scale bar = 5 μm. **E** Representative images, **F** fluorescence intensity of γ-tubulin in MI oocytes in the indicated groups (*n* = 27 per group), and **G** fluorescence intensity of α-tubulin in MI oocytes in the indicated groups (*n* = 39 per group). Scale bar = 50 μm. Quantification of **H** abnormal α-tubulin structures, **I** abnormal chromosomes, **J** lagging chromosomes, **K** spindle width, **L** spindle length, and **M** metaphase plate width in the indicated groups (*n* = 35 per group). **N** Representative images and **O** fluorescence intensity of Ac-tubulin in MI oocytes in the indicated groups (*n* = 50 per group). Scale bar = 50 μm. **P** Representative images and **Q** fluorescence intensity of BUBR1 in MI oocytes in the indicated groups (*n* = 46 per group). Scale bar = 5 μm. **R** qRT-PCR analysis of transcript levels of spindle assembly checkpoint-related genes (*PLK1*, *PP2A*, *PRP4*, *MPS1*, *MAD1*, *MAD2*, *HEC1*, *BUB1*, *BUB3*, *BUBR1*, and *AURKB*) in MI oocytes in the indicated groups (*n* = 3 per group). Data are mean ± SEM for three independent experiments. Different letters indicate significant differences (*P* < 0.05)

To evaluate the effects of NEK2 inhibition on the meiotic cell cycle in porcine oocytes, we analyzed nuclear maturation stages. The majority of control oocytes reached the MII stage by 44 h of IVM, whereas MBM treatment resulted in arrest at MI or incomplete progression to MII (Fig. 3A, B). Chromosomal analysis revealed that NEK2 inhibition significantly increased aneuploidy in MII oocytes compared with controls (Fig. 3C, D). In MI stage oocytes, NEK2 inhibition resulted in reduced γ-tubulin and α-tubulin intensities, accompanied by an increased proportion of abnormal α-tubulin structures (Fig. 3E–H). Spindle damage caused by NEK2 inhibition also increased the incidence of abnormal and lagging chromosomes (Fig. 3I, J). To further investigate these spindle and chromosomal defects, we measured the spindle width, spindle length, and metaphase plate width; all were significantly increased in NEK2-inhibited oocytes compared with control oocytes (Fig. 3K–M). Because microtubule acetylation is essential for spindle stability and assembly, we examined acetylated tubulin levels and found that NEK2 suppression markedly reduced acetylated tubulin intensity relative to the controls (Fig. 3N, O). BUBR1, a core component of the SAC that regulates K–MT attachment and centrosome positioning, was significantly downregulated following NEK2 inhibition. The expression of other SAC-related genes (*PLK1*, *PP2A*, *PRP4*, *MPS1*, *MAD1*, *MAD2*, *HEC1*, *BUB1*, *BUB3*, *BUBR1*, and *AURKB*) was similarly reduced in NEK2-suppressed MI oocytes compared with control MI oocytes (Fig. 3P–R). These findings indicate that NEK2 is critical for proper meiotic cell cycle progression, maintenance of SAC integrity, and microtubule dynamics during porcine oocyte maturation.

### NEK2 regulates the actin network and TZPs in porcine oocyte meiosis

**Fig. 4:**
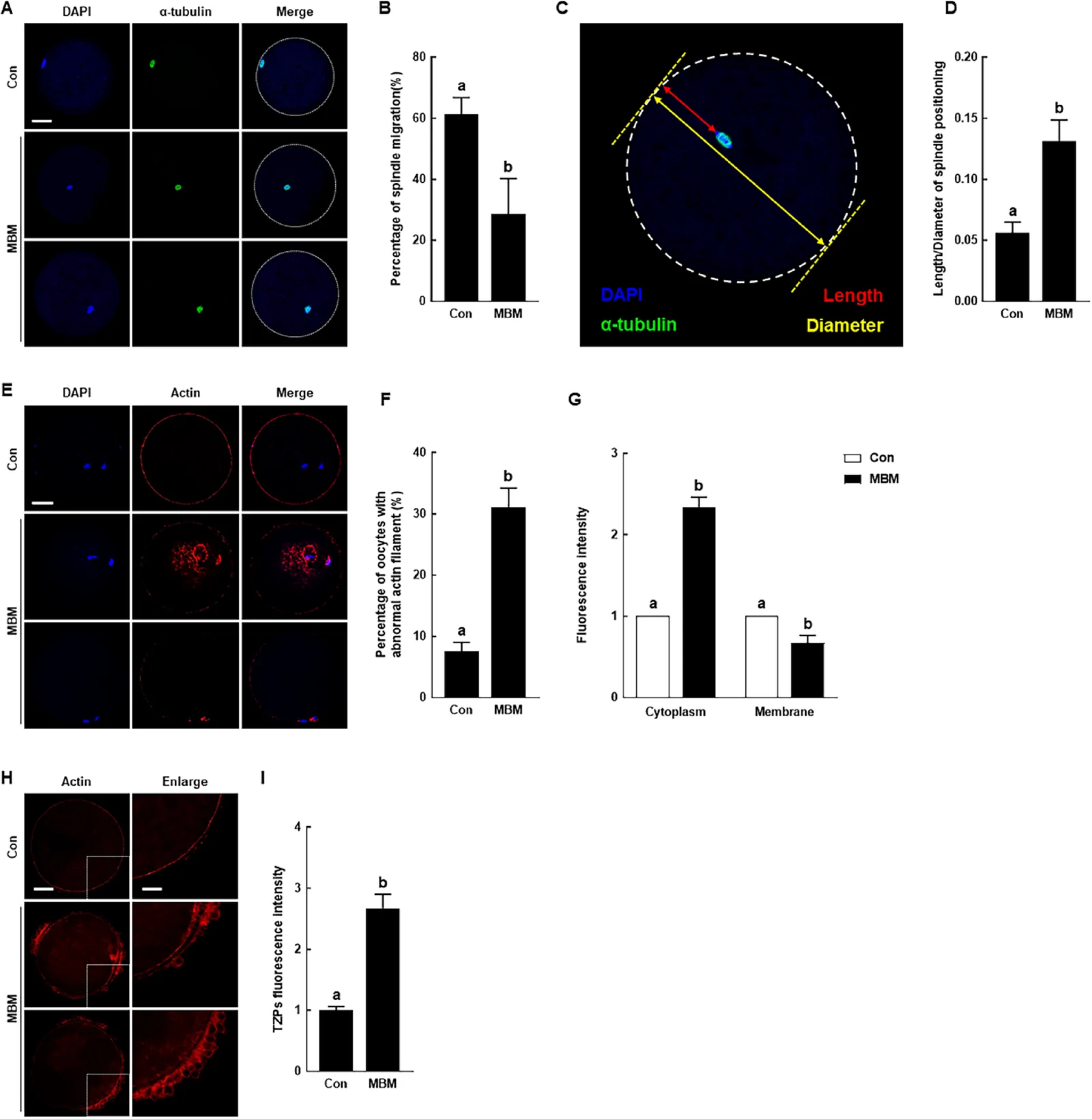
Effects of NEK2 inhibition on spindle positioning, actin, and transzonal projections (TZPs) during oocyte meiotic progression. **A** Representative images and **B** percentage of spindle migration in MI oocytes in the indicated groups (Con: *n* = 57, MBM: *n* = 42). Scale bar = 50 μm. **C** Representative images and **D** spindle positioning in MI oocytes in the indicated groups (*n* = 42 per group). **E** Representative images and **F** percentage of abnormal actin filaments in MII oocytes in the indicated groups (Con: *n* = 68, MBM: *n* = 55), and **G** fluorescence intensity of actin filaments in MII oocytes in the indicated groups (*n* = 59 per group). Scale bar = 50 μm. **H** Representative images and **I** fluorescence intensity of TZPs in MII oocytes in the indicated groups (*n* = 52 per group). Scale bar = 50 μm. Data are mean ± SEM for three independent experiments. Different letters indicate significant differences (*P* < 0.05)

Spindle positioning and actin network activity are essential for regulating asymmetric cell division, which drives polar body extrusion. We investigated the effects of SAC defects induced by NEK2 inhibition on spindle positioning, migration, and actin network organization. Compared with controls, NEK2-inhibited oocytes exhibited reduced spindle migration and impaired positioning (Fig. 4A–D). Moreover, MBM treatment increased the proportion of abnormal actin, elevated cytoplasmic actin intensity, and reduced plasma membrane intensity relative to controls (Fig. 4E–G). Notably, NEK2-inhibited oocytes retained TZPs at a stage when they are typically reduced, resulting in higher TZPs intensity compared with control oocytes (Fig. 4H, I). Collectively, these findings imply that NEK2 is critical for maintaining spindle positioning, actin network, and normal TZPs dynamics during porcine oocyte maturation.

### NEK2 regulates the DNA damage response in porcine oocytes

**Fig. 5:**
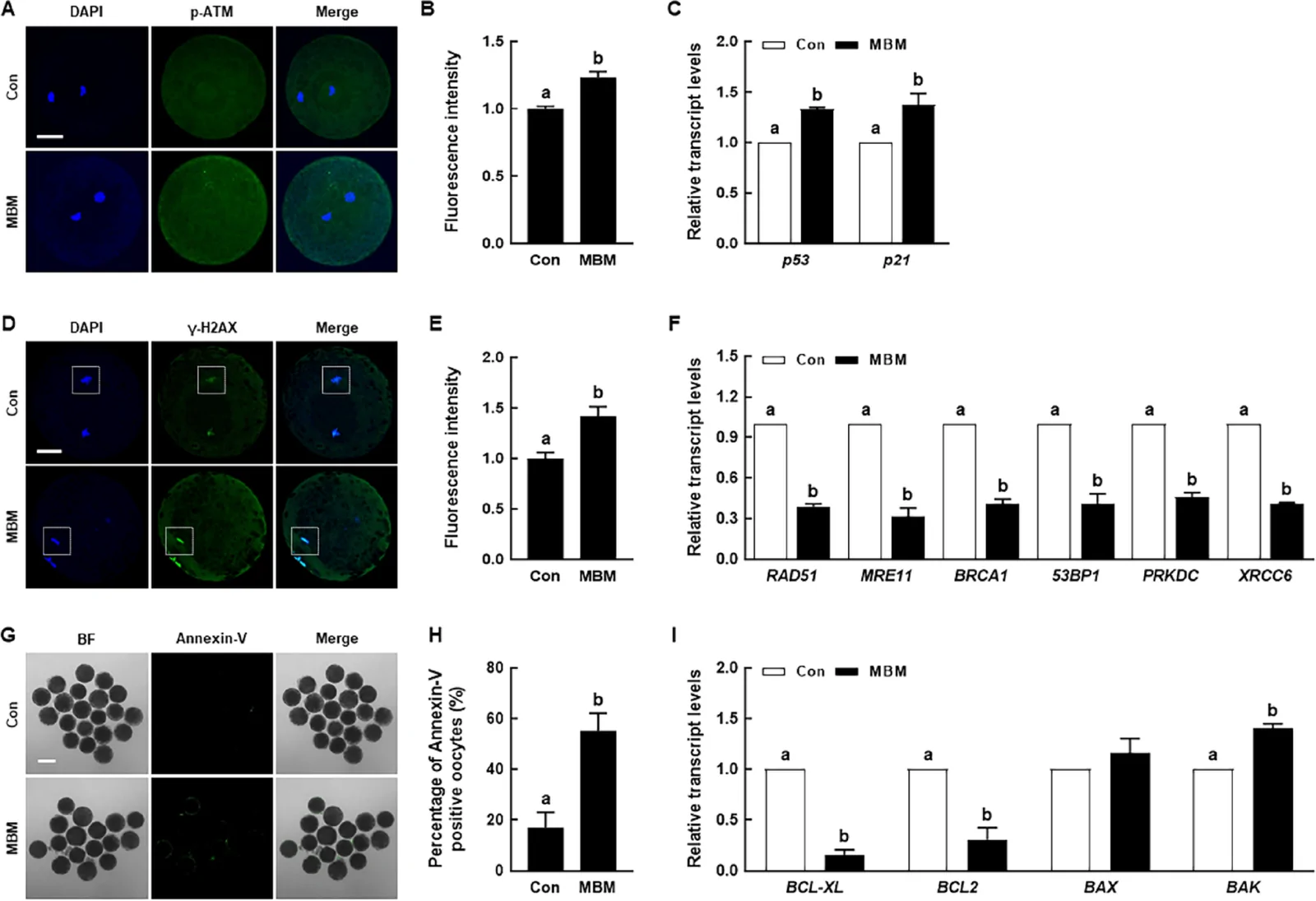
Effects of NEK2 inhibition on DNA damage in porcine oocytes. **A** Representative images and **B** fluorescence intensity of ATM in oocytes in the indicated groups (*n* = 48 per group). Scale bar = 50 μm. **C** qRT-PCR analysis of transcript levels of DNA damage-related genes (*p53*, *p21*) in oocytes in the indicated groups (*n* = 3 per group). **D** Representative images and **E** fluorescence intensity of γ-H2AX in oocytes in the indicated groups (*n* = 55 per group). Scale bar = 50 μm. **F** qRT-PCR analysis of transcript levels of DNA damage repair-related genes (*RAD51*, *MRE11*, *BRCA1*, *53BP1*, *PRKDC*, and *XRCC6*) in oocytes in the indicated groups (*n* = 3 per group). **G** Representative images and **H** percentage of annexin V-positive oocytes in the indicated groups (Con: *n* = 47, MBM: *n* = 41). Scale bar = 100 μm. **I** qRT-PCR analysis of transcript levels of apoptosis-related genes (*BCL-XL*, *BCL2*, *BAX*, and *BAK*) in oocytes in the indicated groups (*n* = 3 per group). Data are mean ± SEM for three independent experiments. Different letters indicate significant differences (*P* < 0.05)

Because NEK2 regulates cellular responses to DNA damage in somatic cells and mammalian embryos, we investigated the effects of NEK2 inhibition on DNA damage levels in porcine oocytes. NEK2-inhibited oocytes showed elevated ATM levels and significantly increased expression of DNA damage-related genes, including *p53* and *p21*, compared with control oocytes (Fig. 5A–C). In addition, NEK2 inhibition led to increased γH2AX levels and significantly decreased the expression of DNA damage repair-related genes (*RAD51*, *MRE11*, *BRCA1*, *53BP1*, *PRKDC*, and *XRCC6*) compared with controls (Fig. 5D–F). DNA damage accumulation also increased the proportion of annexin V-positive oocytes in NEK2-inhibited oocytes compared with control oocytes, a marker of early apoptosis (Fig. 5G–H). Furthermore, compared with control oocytes, anti-apoptotic genes (*BCL-XL* and *BCL2*) were downregulated, while pro-apoptotic genes (*BAX* and *BAK*) were upregulated (Fig. 5I). These findings suggest that NEK2 inhibition induces DNA damage, which culminates in porcine oocyte apoptosis due to insufficient repair.

### Inhibition of NEK2 during IVM reduces embryo development competence

**Fig. 6:**
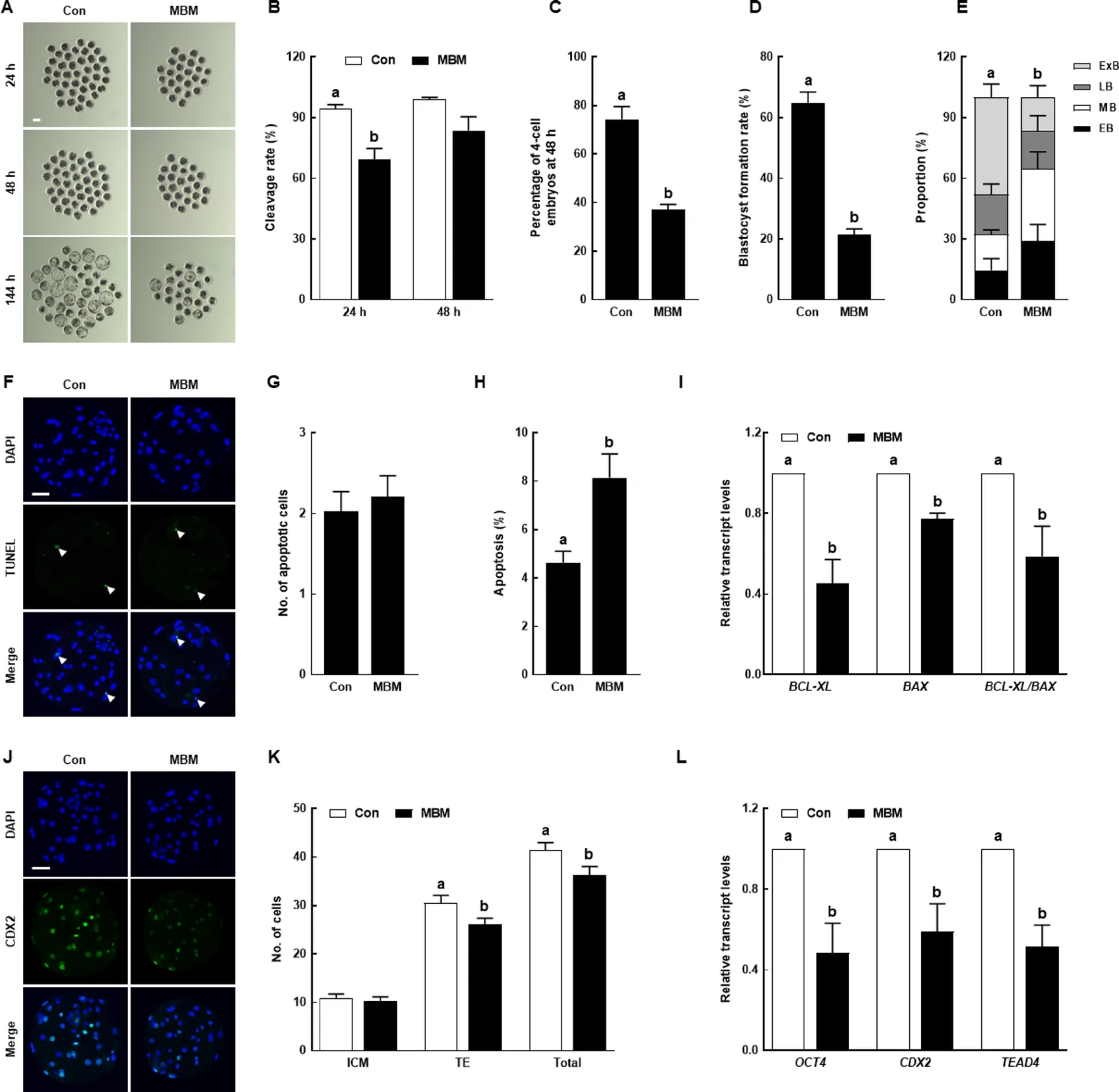
Effects of NEK2 inhibition during IVM on porcine embryogenesis. **A** Representative images of cleavage and embryonic development, **B** cleavage rate, **C** percentages of four-cell embryos, and **D** blastocyst formation rates in the indicated groups (Con: *n* = 127, MBM: *n* = 68). Scale bar = 100 μm. **E** Proportions of different blastocyst stages in the indicated groups (Con: *n* = 93, MBM: *n* = 23). **F** Representative images, **G** number of apoptotic cells, and **H** apoptosis rate in blastocysts in the indicated groups (*n* = 38 per group). Scale bar = 50 μm. **I** qRT-PCR analysis of transcript levels of apoptosis-related genes (*BCL-XL*, *BAX*) in blastocysts in the indicated groups (*n* = 3 per group). **J** Representative images and **K** number of inner cell mass, trophectoderm, and total cells in blastocysts in the indicated groups (*n* = 33 per group). Scale bar = 50 μm. **L** qRT-PCR analysis of transcript levels of development-related genes (*OCT4*, *CDX2*, and *TEAD4*) in blastocysts in the indicated groups (*n* = 3 per group). Data are mean ± SEM for three independent experiments. Different letters indicate significant differences (*P* < 0.05)

We evaluated the embryonic developmental capacity of porcine oocytes following NEK2 inhibition during IVM. NEK2 inhibition during IVM significantly reduced the cleavage rate at 24 h and the four-cell embryo rate at 48 h compared with controls (Fig. 6A–C and Supplementary Table 3). MBM treatment decreased the blastocyst formation rate and reduced the proportion of expanded blastocysts compared with controls (Fig. 6D, E and Supplementary Tables 3, 4). An examination of blastocyst quality showed that, compared with controls, NEK2 inhibition during IVM significantly increased the apoptosis rate and altered the expression of apoptosis-related genes *(BCL-XL* and *BAX*), resulting in a decreased *BCL-XL*/*BAX* ratio in NEK2 inhibition (Fig. 6F–I and Supplementary Table 5). It also reduced the number of trophectoderm cells and total cells, as well as the expression of development-related genes (*OCT4*, *CDX2*, and *TEAD4*) under NEK2 inhibition compared with controls (Fig. 6J–L and Supplementary Table 6). These results suggest that NEK2 plays critical roles in oocyte maturation and embryonic development.

## Discussion

**Fig. 7:**
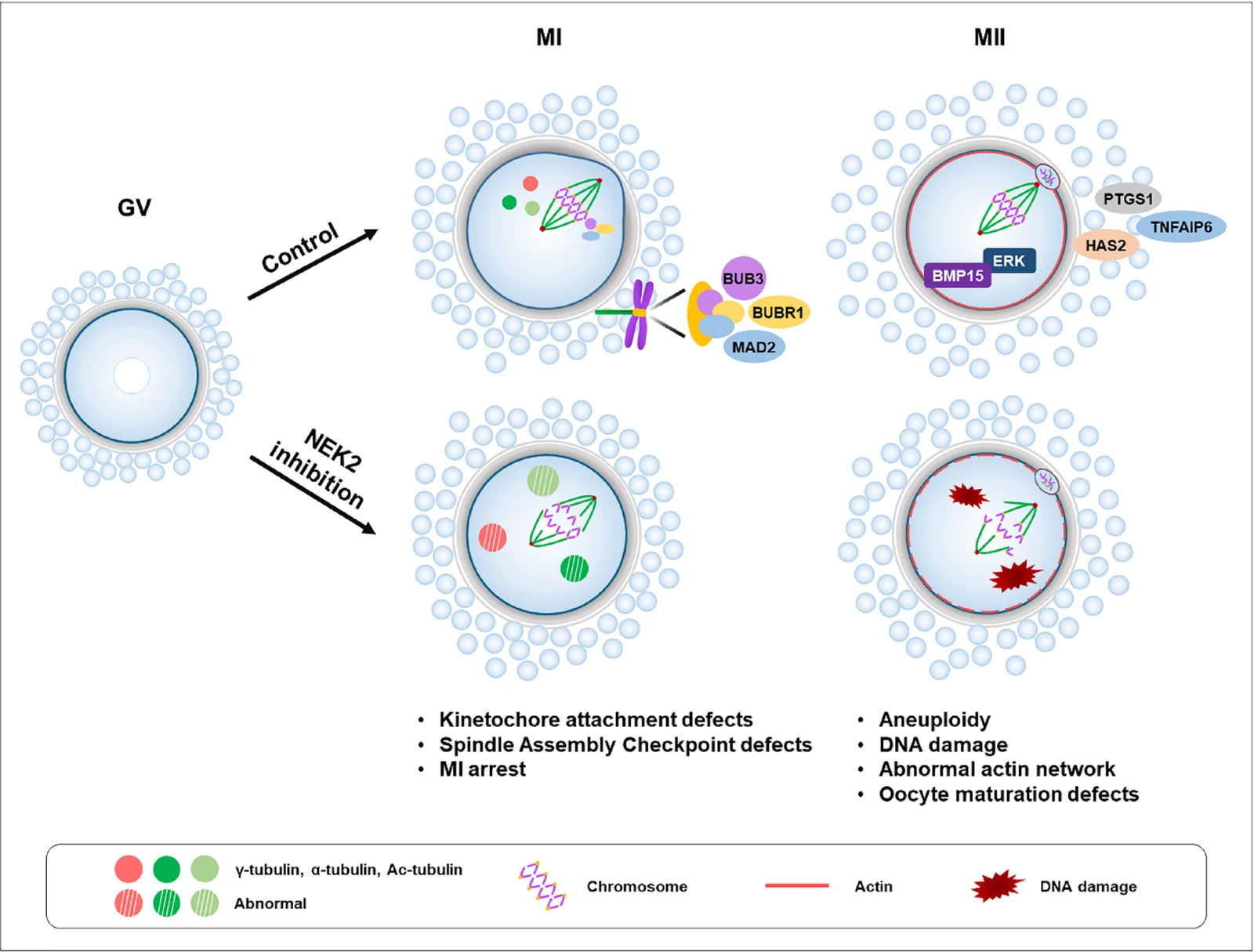
Graphical overview of the effects of NEK2 inhibition on porcine oocyte maturation. NEK2 maintains spindle assembly checkpoint integrity by regulating microtubule stability and kinetochore–microtubule attachment during meiotic maturation in porcine oocytes. NEK2 also contributes to actin network organization and proper DNA damage response. Through these coordinated mechanisms, NEK2 ensures the maintenance of euploidy and supports successful oocyte maturation

NEK2 is a multifunctional protein in the NIMA-related kinase family that plays essential roles in centrosome separation, microtubule stabilization, cell cycle regulation, and the DNA damage response [20]. However, its specific role in porcine oocyte meiotic maturation has remained poorly understood. This study investigated the influence of NEK2 on oocyte maturation, SAC function, cytoskeletal dynamics, and the DNA damage response. By pharmacologically inhibiting NEK2, we demonstrated that this kinase is indispensable for maintaining euploidy, microtubule stability, K–MT attachment, SAC integrity, actin network organization, and sufficient DNA damage response in porcine oocytes during meiotic maturation (Fig. 7). Because meiotic maturation depends on the accumulation of various maternal mRNAs and proteins, characterizing oocyte gene expression is crucial for understanding the regulation of oocyte maturation, fertility, and subsequent embryonic development [21]. NEK2 exists in three major isoforms and regulates diverse cellular processes including centrosome duplication, spindle organization, and chromosome segregation [22, 23]. Our findings demonstrated that NEK2 is abundantly expressed in porcine oocytes and cumulus cells, and exhibits a broad distribution throughout the nucleus and cytoplasm during oocyte maturation. The elevated NEK2 mRNA levels observed at the MI stage suggest a potential involvement of NEK2 in regulating chromosome dynamics during meiosis. Our findings are partially consistent with a previous study which reported constitutive NEK2 expression throughout mouse oocyte maturation [4], further supporting a potential role for NEK2 in mammalian oocyte maturation. To further investigate whether NEK2 acts as a key regulator of porcine oocyte maturation, we inhibited NEK2 activity using a specific inhibitor. Because normal cumulus cell function and cumulus–oocyte communication are essential for oocyte maturation, we additionally investigated the effects of NEK2 inhibition on both cumulus cells and oocytes to evaluate their potential contribution to oocyte maturation competence. NEK2 inhibition significantly reduced the rate of polar body extrusion and partially impaired the cell cycle in cumulus cells, accompanied by impaired cumulus expansion, with most NEK2-inhibited oocytes arrested at the MI stage, consistent with the elevated NEK2 mRNA expression observed at this phase. NEK2 inhibition during the meiotic maturation of porcine oocytes impaired subsequent embryonic development. Furthermore, NEK2 inhibition increased apoptosis rates and decreased total cell numbers in blastocysts. These findings suggest that proper regulation of NEK2 activity during oocyte meiotic maturation is essential for normal embryonic development, and highlight the necessity of NEK2 activity for successful progression through meiotic maturation in porcine oocytes. The results warrant further investigation into the underlying molecular mechanisms during the MI stage.

During the MI stage of oocyte maturation, the spindle plays a crucial role in ensuring accurate chromosome alignment and segregation through processes regulated by the aMTOCs. The aMTOCs organizes spindle fibers that attach to chromosomes via kinetochores, thereby ensuring proper chromosome alignment and asymmetric division. This process, which is closely associated with cytoskeletal integrity, is essential for euploid nucleus formation [24, 25]. Microtubule-associated proteins are key regulators of spindle assembly and chromosome segregation during meiotic maturation [26]. Among these, γ-tubulin, a component of the pericentriolar material within the aMTOCs, contributes to microtubule nucleation and centrosome maturation [27, 28]. Previous studies have reported that NEK2 facilitates γ-tubulin recruitment in *Xenopus* and that its activity is tightly associated with pericentriolar material protein regulation [14, 29]. Moreover, NEK2 inhibition by dsRNA induces abnormal spindle architecture in mouse oocytes [4]. NEK2 promotes centrosome separation by phosphorylating centrosomal proteins such as C-NAP1 and rootletin, which are important in spindle assembly processes [30]. Since proper aMTOC organization and spindle assembly are essential for establishing stable chromosome–spindle interactions, disruption of NEK2 activity may indirectly impair K–MT attachment stability through abnormalities in microtubule organization and spindle dynamics. Consistent with this notion, our study demonstrated that NEK2 inhibition induces severe spindle abnormalities in porcine oocytes, leading to increased chromosome misalignment. This phenotype likely stems from compromised aMTOC function driven by reduced γ-tubulin levels, and is further exacerbated by decreased microtubule stability, as reflected by lower Ac-tubulin expression. Given that stable K–MT interactions are indispensable for accurate chromosome alignment and subsequent segregation, the disruption of spindle organization and microtubule dynamics under NEK2 inhibition collectively culminates in impaired chromosome–spindle attachment stability during meiotic progression. The SAC is essential for accurate chromosome segregation, and HEC1 contributes to faithful chromosome segregation and maintenance of genomic integrity by mediating spindle checkpoint signaling and chromosome alignment. The core components of the SAC include several checkpoint proteins, including members of the MAD and BUB families, as well as MPS1 [20]. Previous studies have reported that NEK2 contributes to chromosome segregation by directly phosphorylating the S165 residue of HEC1 and by interacting with MAD1, thereby promoting proper MAD2 recruitment to kinetochores and maintaining spindle checkpoint signaling [31, 32]. Consistent with this possibility, NEK2 inhibition in the present study resulted in reduced BUBR1 localization together with decreased expression of multiple SAC-related genes, including members of the MAD family, HEC1, and BUB family genes, suggesting that SAC dysregulation during porcine oocyte maturation may be associated with alterations in kinetochore-associated signaling rather than the direct regulation of individual SAC components. 

Actin is a major cytoskeletal element in oocytes that is involved in multiple biological processes during maturation, including spindle migration, cortical formation, and polarity establishment [33]. During oocyte maturation, the spindle must translocate toward the cortex in an actin-dependent manner, in a process mediated by the chromosome–spindle complex [26, 34]. Actin-associated proteins accumulate in the oocyte cortex at the MI and MII stages, overlapping with F-actin distribution, where actin filaments cooperate with microtubules to mediate centrosome separation [35–37]. GAS2L1, a protein that binds microtubules and F-actin, is phosphorylated by NEK2, which in turn stimulates F-actin activity [38]. NEK2 has also been reported to interact with and phosphorylate RhoGDI1, a key regulator of actin cytoskeletal rearrangement [39]. Thus, NEK2 is an important modulator of actin dynamics through multiple mechanisms. In this study, NEK2 inhibition reduced spindle migration, altered cytoskeletal organization, decreased cortical actin intensity, and increased abnormal actin distribution. In addition to its role in regulating actin organization within the oocyte, NEK2 may also influence actin-dependent communication between the oocyte and surrounding cumulus cells. Because TZPs are predominantly composed of F-actin-based filaments, their formation, maintenance, and remodeling are closely dependent on actin cytoskeletal dynamics [40, 41]. Therefore, alterations in actin organization following NEK2 inhibition may influence TZP structure and remodeling during oocyte maturation. TZPs are specialized membrane structures of cumulus cells that connect to the oocyte and are responsible for the exchange of information and materials between them [42]. Through these connections, oocytes regulate the expansion, proliferation, differentiation, and apoptosis of surrounding cumulus cells. In addition, TZPs facilitate the transfer of metabolites, signaling molecules, and regulatory factors required for proper oocyte growth and acquisition of developmental competence [40]. During oocyte maturation, TZPs, which are primarily composed of F-actin, are gradually degraded as cumulus cells expand [41]. Proper remodeling of TZPs is considered an important event during meiotic progression because it coordinates communication between cumulus cells and oocytes and supports normal maturation of the cumulus–oocyte complex. Our findings suggest that NEK2 inhibition delays the timely depletion of TZPs, possibly by disrupting actin organization and remodeling, thereby interfering with normal cumulus–oocyte interactions and contributing to impaired oocyte maturation.

Defects in the SAC prevent the efficient repair of damaged DNA. Furthermore, abnormal microtubule and actin networks contribute to the accumulation of DNA damage [43]. Gene set enrichment analysis has shown that NEK2 is highly correlated with the mismatch repair and nucleotide excision repair pathways, which suggests that NEK2 is involved in the DNA damage response [44]. NEK2 regulates p53-mediated apoptosis and p53 phosphorylation at Ser315, and participates in DNA damage repair through various signaling pathways, including Wnt/β-catenin and AKT [19, 45, 46]. NEK2 inhibition accelerated DNA damage accumulation during porcine embryogenesis, activating the ATM–p53–p21 pathway and reducing the expression of genes associated with HR and NHEJ [18]. Consistent with these observations, which demonstrate that NEK2 plays a regulatory role in the DNA repair system, our study revealed that NEK2 inhibition increased DNA damage in porcine oocytes, accompanied by activation of the ATM–p53–p21 pathway and elevated γH2AX levels. Furthermore, the accumulation of DNA damage suppressed activation of the HR and NHEJ pathways, thereby inducing apoptosis. Therefore, NEK2 is essential for maintaining genomic stability by regulating the DNA damage response during meiosis in porcine oocytes.

Oocyte quality directly influences fertilization and subsequent embryonic development, and is closely associated with molecular processes such as mRNA storage and translation, which regulate developmental competence [47]. Both nuclear and cytoplasmic maturation are essential prerequisites for successful embryonic development and are fundamental determinants of blastocyst quality. During IVM, MBM-mediated inhibition of NEK2 impaired normal oocyte maturation, which in turn negatively affected subsequent embryonic development, resulting in lower blastocyst formation rates and reduced blastocyst quality. Thus, NEK2 plays critical roles in oocyte maturation by coordinating key processes in both the nucleus and cytoplasm, including proper chromosome–spindle organization, SAC regulation, actin dynamics, and cumulus cell–oocyte communication. While the phenotypes observed in the present study are consistent with previously reported functions of NEK2, interpretation of these findings should also consider the inherent limitations associated with pharmacological inhibition approaches.

## Conclusion

This study is the first to demonstrate that NEK2 plays critical roles in K–MT attachment, SAC function, and cytoskeletal dynamics during porcine oocyte maturation. Inhibition of NEK2 led to defective K–MT attachment, abnormal spindle morphology, and chromosome misalignment, along with reduced BUBR1 levels, indicating SAC dysfunction. Furthermore, NEK2 inhibition decreased the motility of the chromosome–spindle complex and caused abnormal actin distribution due to impaired actin dynamics. Therefore, our findings identify NEK2 as a key regulatory gene involved in oocyte meiotic maturation, fertility, and subsequent embryonic development.

## Supplementary Information

Below is the link to the electronic supplementary material.

Supplementary file1 (DOCX 30 KB)

## Data Availability

All data supporting the findings of this study are available within the paper and its Supplementary Information.
