# Supplementary material for: NEK2 is essential for spindle assembly checkpoint regulation and cytoskeleton organization in porcine oocyte meiotic maturation
Source: Mol Cell Biochem. 2026 Aug 4;481(9):4549–64. doi: 10.1007/s11010-026-05674-z (PMC13638756; doi:10.1007/s11010-026-05674-z)
Supplement: Supplementary file 1 [file 11010_2026_5674_MOESM1_ESM.docx]

Supplementary Table 1. Primer sequences for qRT-PCR

| Gene | Primer sequences | GenBank  accession no. | Product  size (bp) |
| --- | --- | --- | --- |
| *NEK2* | F: 5’- CACTTCAGCGGGGAGAGTAA − 3’ | XM_021063863.1 | 100 |
|  | R: 5’- GGAGCCTCCTCTTCAGGTCT − 3’ |  |  |
| *PTGS1* | F: 5’- CAACACGGCACACGACTACA − 3’ | XM_001926129.6 | 121 |
|  | R: 5’- CTGCTTCTTCCCTTTGGTCC − 3’ |  |  |
| *TNFAIP6* | F: 5’- AGAAGCGAAAGATGGGATGCT − 3’ | NM_001159607.1 | 106 |
|  | R: 5’- CATTTGGGAAGCCTGGAGATT − 3’ |  |  |
| *HAS2* | F: 5’- AGTTTATGGGCAGCCAATGTAGTT − 3’ | AB050389 | 101 |
|  | R: 5’- GCACTTGGACCGAGCTGTGT − 3’ |  |  |
| *BAX* | F: 5’- CGATCTCGAAGGAAGTCCAG − 3’ | XM_003127290.5 | 251 |
|  | R: 5’- AAGCGCATTGGAGATGAACT − 3’ |  |  |
| *BXL-XL* | F: 5’- AGGGCATTCAGTGACCTGAC − 3’ | NM_214285.1 | 242 |
|  | R: 5’- TGGATCCAAGGCTCTAGGTG − 3’ |  |  |
| *BCL2* | F: 5’- GGAGGGGACACTCTTCTTCC − 3’ | XM_021099593.1 | 189 |
|  | R: 5’- CTGGGCACAATTGGTAGCTT − 3’ |  |  |
| *BAK* | F: 5’- CTAGAACCTAGCAGCACCAT − 3’ | XM_021098604.1 | 115 |
|  | R: 5’- CGATCTTGGTGAAGTACTC − 3’ |  |  |
| *BMP15* | F: 5’- TGGTGAGGCCATTGGTTAAT − 3’ | NM_001005155.2 | 156 |
|  | R: 5’- AGAGGTGGAAGGGAGCTAGG − 3’ |  |  |
| *GDP9* | F: 5’- AACACTGTCCGGCTCTTCAC − 3’ | NM_001001909.1 | 202 |
|  | R: 5’- CCAGGCTGCACTCACATTTA − 3’ |  |  |
| *CylinB1* | F: 5’- TGTGTGCCCAAGAAGATGCT − 3’ | NM_001170768.1 | 189 |
|  | R: 5’- AGGGCGACCCAGACAAAAAT − 3’ |  |  |
| *C-MOS* | F: 5’- ATCATCATGGAGTTCGGCGG − 3’ | NM_001113219.1 | 113 |
|  | R: 5’- TCCAAGTTCAGTTGCTCCCC − 3’ |  |  |
| *CDK1* | F: 5’- CCAGAAATTCGCTTGGCAGG − 3’ | NM_001159304.2 | 157 |
|  | R: 5’- AAGTGTGGCCAGAAGTGGAG − 3’ |  |  |
| *PLK1* | F: 5’- CGAGTCCCTGTCAATCAAGC − 3’ | XM_021086465.1 | 110 |
|  | R: 5’- TTAAAATCCAAACCCGCCCG − 3’ |  |  |
| *PP2A* | F: 5’- GTGGCATCATGGACGAGAAG − 3’ | NM_214366.1 | 156 |
|  | R: 5’- ATCGAACCTCTTGCACGTTG − 3’ |  |  |
| *PRP4* | F: 5’- AGCCCGGCAAATCAATGTTT − 3’ | XM_005660339.3 | 103 |
|  | R: 5’- TCTTTCAGCAGGACCCTCTC − 3’ |  |  |
| *MPS1* | F: 5’- TGCATTCTCTCCAGTAGCGA − 3’ | XM_021092077.1 | 110 |
|  | R: 5’- AGACACCACAGCACATCTACT − 3’ |  |  |
| *MAD1* | F: 5’- CATTTGGGGCTGAGGAATCG − 3’ | XM_021085975.1 | 122 |
|  | R: 5’- TTACGAGTCACTGTGGAGGG − 3’ |  |  |
| *MAD2* | F: 5’- TACTTTTGAAACCGGTGGCG − 3’ | XM_003129223.4 | 130 |
|  | R: 5’- GGTAATGCCTTGCTCCCGG − 3’ |  |  |
| *HEC1* | F: 5’- CGAAGTTCAGTTTCCACCGG − 3’ | XM_021096139.1 | 101 |
|  | R: 5’- TCTTTGGTTTGAGGGGTATGGA − 3’ |  |  |
| *BUB1* | F: 5’- CCCCTGAGAAAGTGATGCCT − 3’ | XM_021087047.1 | 110 |
|  | R: 5’- TGGCTTGATGTCTCCATGGA − 3’ |  |  |
| *BUB3* | F: 5’- TGACCGGTTCTAACGAGTTCA − 3’ | XM_001928045.3 | 110 |
|  | R: 5’- TGTGTCCCAGGAAGAAACCA − 3’ |  |  |
| *BUBR1* | F: 5’- AGGAGGACGAAGCTTTTGGA − 3’ | XM_021097824.1 | 105 |
|  | R: 5’- CTCCTACACGGCTGATTGGA − 3’ |  |  |
| *AURKB* | F: 5’- ATTTCATCGTGGCGCTCAAA − 3’ | XM_021066198.1 | 111 |
|  | R: 5’- TGTTTGGATGCTGCAGATGG − 3’ |  |  |
| *p53* | F: 5’- CCTCACCATCATCACACTGG − 3’ | NM_213824.3 | 214 |
|  | R: 5’- GGCTTTCTTCTTTTGCACTGG − 3’ |  |  |
| *p21* | F: 5’- CCCCTCTGTCCAGTGAAAGT − 3’ | XM_013977858.2 | 103 |
|  | R: 5’- CCAGAAAAGTGCAGGGGAAG − 3’ |  |  |
| *RAD51* | F: 5’- CTGCAGGCCGAGTATTGAAC − 3’ | NM_001123181.1 | 125 |
|  | R: 5’- TGCCACATTGCTCTAATCGT − 3’ |  |  |
| *MRE11* | F: 5’- CAAGAGGCCATGAACAGAGC − 3’ | XM_003129788.5 | 107 |
|  | R: 5’- GCCGTCTGTTCTGCCATATC − 3’ |  |  |
| *BRCA1* | F: 5’- ACCCTGAGTCTGATCCCTCT − 3’ | XM_021066931.1 | 125 |
|  | R: 5’- AGCTGGACTCTTGGCAGATT − 3’ |  |  |
| *53BP1* | F: 5’- AACGGCGAAGTAACATCAGC − 3’ | XM_021096915.1 | 100 |
|  | R: 5’- GTGGAAGCACGAGGACTTTC − 3’ |  |  |
| *PRKDC* | F: 5’- ATCCTTGGCAGAACTTGGGA − 3’ | XM_021089419.1 | 110 |
|  | R: 5’- CGGATCGCTCAAAGTTCAGG − 3’ |  |  |
| *XRCC6* | F: 5’- TTCCTGGACTTGATGCACCT − 3’ | NM_001190185.1 | 115 |
|  | R: 5’- GCGACTCCTCAAAGTGAACC − 3’ |  |  |
| *OCT4* | F: 5’- AGTGAGAGGCAACCTGGAGA − 3’ | NM_001113060.1 | 151 |
|  | R: 5’- ACTGCTTGATCGTTTGCCCT − 3’ |  |  |
| *CDX2* | F: 5’- GGCAGCCAAGTGAAAACCAG − 3’ | NM_001278769.1 | 119 |
|  | R: 5’- GCCTTTCTCCGAATGGTGAT − 3’ |  |  |
| *TEAD4* | F: 5’- GATGTACGGTCGGAATGAG − 3’ | NM_001142666 | 145 |
|  | R: 5’- TGCCTGATCCTTTAGCTTGG − 3’ |  |  |
| *GAPDH* | F: 5’- TCGGAGTGAACGGATTTGGC − 3’ | NM_001206359.1 | 147 |
|  | R: 5’- TGCCGTGGGTGGAATCATAC − 3’ |  |  |

F: forward, R: reverse

Supplementary Table 2. Effects of NEK2 inhibition on nuclear maturation of porcine oocytes

| MBM-55 (µM) | No. of oocytes examined | Metaphase II (%) | Immature (%) | Degenerate (%) |
| --- | --- | --- | --- | --- |
| 0 | 200 | 172 (86.0 ± 2.4)^a^ | 13 (6.5 ± 0.5)^a^ | 15 (7.5 ± 2.7) ^a^ |
| 6 | 200 | 148 (74.0 ± 2.1) ^a^ | 37 (18.5 ± 0.9)^b^ | 15 (7.5 ± 1.7) ^a^ |
| 8 | 200 | 109 (54.5 ± 3.9)^c^ | 77 (38.5 ± 2.9)^c^ | 14 (7.0 ± 1.7) ^a^ |
| 10 | 200 | 39 (19.5 ± 1.7)^d^ | 122 (61.0 ± 2.8)^d^ | 39 (19.5 ± 2.7) ^a^ |

Data are the mean ± SEM, and values with different superscript letter within a column differ significantly (*P* < 0.05).

Supplementary Table 3. Effects of NEK2 inhibition during *in vitro* maturation on *in vitro* development of porcine parthenogenetic embryos

| Groups | No. of  embryos examined | Cleavage (%) | | Blastocyst (%) |
| --- | --- | --- | --- | --- |
|  |  | 24 h | 48 h |  |
| Con | 200 | 120 (94.6 ± 1.8)^a^ | 126 (99.1 ± 0.8) | 82 (64.8 ± 3.6)^a^ |
| MBM | 200 | 49 (69.0 ± 5.7)^b^ | 59 (83.7 ± 6.7) | 14 (21.5 ± 1.8)^b^ |

Data are the mean ± SEM, and values with different superscript letter within a column differ significantly (*P* < 0.05).

Supplementary Table 4. Effects of NEK2 inhibition during *in vitro* maturation on the post-blastulation development of porcine parthenogenetic blastocysts

| Groups | No. of  blastocysts examined | Proportion of blastocysts developed to the following stages (%) | | | |
| --- | --- | --- | --- | --- | --- |
|  |  | Early | Middle | Large | Expanded |
| Con | 93 | 14.5 ± 5.7 | 17.7 ± 2.2 | 19.6 ± 5.3 | 48.0 ± 6.6^a^ |
| MBM | 23 | 28.9 ± 8.2 | 35.8 ± 8.2 | 18.5 ± 7.6 | 16.6 ± 5.8^b^ |

Data are the mean ± SEM, and values with different superscript letter within a column differ significantly (*P* < 0.05).

Supplementary Table 5. Effects of NEK2 inhibition during *in vitro* maturation on cell survival in porcine parthenogenetic blastocysts

| Groups | No. of blastocysts examined | No. of TUNEL-positive cells | Apoptosis (%) |
| --- | --- | --- | --- |
| Con | 38 | 2.0 ± 0.2 | 4.6 ± 0.4^a^ |
| MBM | 38 | 2.2 ± 0.2 | 8.1 ± 0.9^b^ |

Data are the mean ± SEM, and values with different superscript letter within a column differ significantly (*P* < 0.05).

Supplementary Table 6. Effects of NEK2 inhibition during *in vitro* maturation on the inner cell mass (ICM) and trophectoderm (TE) cell number in porcine parthenogenetic blastocysts

| Groups | No. of blastocysts examined | No. of nuclei | | |
| --- | --- | --- | --- | --- |
|  |  | ICM | TE | Total |
| Con | 33 | 10.8 ± 0.8 | 30.4 ± 1.6^a^ | 41.3 ± 1.6^a^ |
| MBM | 33 | 10.2 ± 0.8 | 26.0 ± 1.2^b^ | 36.3 ± 1.8^b^ |

Data are the mean ± SEM, and values with different superscript letter within a column differ significantly (*P* < 0.05).
